# Protective Effects of Long-Term Escitalopram Administration on Memory and Hippocampal *BDNF* and *BCL-2* Gene Expressions in Rats Exposed to Predictable and Unpredictable Chronic Mild Stress

**DOI:** 10.3390/brainsci14050420

**Published:** 2024-04-25

**Authors:** Vajihe Saedi Marghmaleki, Maryam Radahmadi, Hojjatallah Alaei, Hossein Khanahmad

**Affiliations:** 1 Department of Physiology, School of Medicine, Isfahan University of Medical Sciences, Isfahan 81746-73461, Iran; vajihe.saedi@yahoo.com; 2 Department of Genetics and Molecular Biology, School of Medicine, Isfahan University of Medical Sciences, Isfahan 81746-73461, Iran; h_khanahmad@med.mui.ac.ir

**Keywords:** escitalopram, predictable stress, unpredictable stress, memory, *BDNF*, *BCL-2*

## Abstract

Stress and escitalopram (an anti-stress medication) can affect brain functions and related gene expression. This study investigated the protective effects of long-term escitalopram administration on memory, as well as on hippocampal *BDNF* and *BCL-2* gene expressions in rats exposed to predictable and unpredictable chronic mild stress (PCMS and UCMS, respectively). Male rats were randomly assigned to different groups: control (Co), sham (Sh), predictable and unpredictable stress (PSt and USt, respectively; 2 h/day for 21 consecutive days), escitalopram (Esc; 10 mg/kg for 21 days), and predictable and unpredictable stress with escitalopram (PSt-Esc and USt-Esc, respectively). The passive avoidance test was used to assess behavioral variables. The expressions of the *BDNF* and *BCL-2* genes were assessed using real-time quantitative PCR. Latency significantly decreased in the PSt and USt groups. Additionally, latency showed significant improvement in the PSt-Esc group compared to the PSt group. The expression of the *BDNF* gene significantly decreased only in the USt group. *BDNF* gene expression significantly increased in the PSt-Esc and USt-Esc groups compared to their respective stress-related groups, whereas the expression of the *BCL-2* gene did not change significantly in both PSt-Esc and USt-Esc groups. PCMS and UCMS had devastating effects on memory. Escitalopram improved memory only under PCMS conditions. PCMS and UCMS exhibited fundamental differences in hippocampal *BDNF* and *BCL-2* gene expressions. Furthermore, escitalopram increased hippocampal *BDNF* gene expression in the PCMS and UCMS subjects. Hence, neurogenesis occurred more significantly than anti-apoptosis under both PCMS and UCMS conditions with escitalopram.

## 1. Introduction

Nowadays, a significant number of individuals are confronted with daily exposure to elevated levels of chronic predictable and unpredictable mild stress (PCMS and UCMS, respectively) [1]. Chronic stress triggers the release of glucocorticoids from adrenal glands [2]. Nevertheless, stress can affect various brain functions, such as learning, memory, and memory consolidation; therefore, cognitive disorders can result from prolonged stress [3,4]. Hence, different forms of stress affect various types of memory [5].

Some stress-related disorders may require alternative treatments and complementary medications [6]. There are several approaches to treating chronic stress disorders, such as chemical antidepressants, herbal plant drugs, and lifestyle changes [7,8]. Changes in the levels of neurotransmitters, such as serotonin, have been found to have a notable impact on the pathophysiology of stress [9]. In this context, serotonin is a vital neurotransmitter that significantly impacts the regulation of both mood and cognitive disorders [10]. Serotonin also regulates other neurotransmitters, such as GABA and glutamate, that are involved in mood and cognition [11,12]. Selective serotonin reuptake inhibitors (SSRIs), such as escitalopram, may reverse learning and memory impairments [13]. However, the negative effects of escitalopram have been observed on cognitive function [14,15].

The hippocampus has been identified as the primary memory region in the brain [16], responsible for synaptic flexibility, the HPA axis, brain mediators, and gene expression [17,18]. Therefore, both chronic stress and escitalopram can impact the structure and function of the brain [19,20]. In contrast, the brain-derived neurotrophic factor (*BDNF*) and B-cell lymphoma (*BCL-2*) genes are involved in neurogenesis (the growth and survival of new neurons) and anti-apoptosis (preventing cellular self-destruction) of the hippocampus, respectively [21,22,23]. *BDNF* achieves this by activating various cellular pathways that support the development and function of neurons. *BCL-2* acts as a shield, protecting neurons from self-destruction. Moreover, some connections between *BDNF* and *BCL-2* are understood in terms of the interplay between them. *BDNF* and BCL-2 work together to enhance the quality of the neural system. *BDNF* might influence the expression of *BCL-2*, promoting the survival of newly generated neurons. Studies have shown that *BDNF* can prevent the activation of caspases, enzymes that dismantle cells during apoptosis, possibly by regulating proteins, such as *BCL-2* [24]. The balance between apoptosis and neurogenesis is crucial for cell turnover in the hippocampus [25]. PCMS and UCMS can damage the hippocampus and impair certain brain functions [26,27]. It has been reported that *BDNF* may mediate brain function under PCMS and UCMS conditions [28,29], as well as change during treatment with antidepressants [30]. In addition, predictable chronic stress exerts a significant impact on the expression of the *BCL-2* gene in hippocampal neurons as a compensatory response [30]. As mentioned earlier, nowadays, a significant number of individuals are confronted with daily exposure to elevated levels of PCMS and UCMS, which can affect brain function differently, including memory. In addition, anti-anxiety medications are frequently used in human societies due to the high levels of stressful events in life and the demands of many stressful jobs (e.g., firefighters, emergency medical jobs, aviation watchtowers, and skin-divers) that require focus on work and the enhancement of brain functions, such as memory. However, escitalopram is administered at a similar dose in clinical settings regardless of the type of stress experienced (predictable or unpredictable). This approach may influence the neural mechanisms and gene expressions involved in memory. Despite extensive research on escitalopram, no published reports have been found on the effects of long-term escitalopram administration on memory and on hippocampal BDNF and BCL-2 gene expressions in rats exposed to predictable and unpredictable chronic mild stress.

## 2. Materials and Methods

### 2.1. Animals

**Figure 1:**
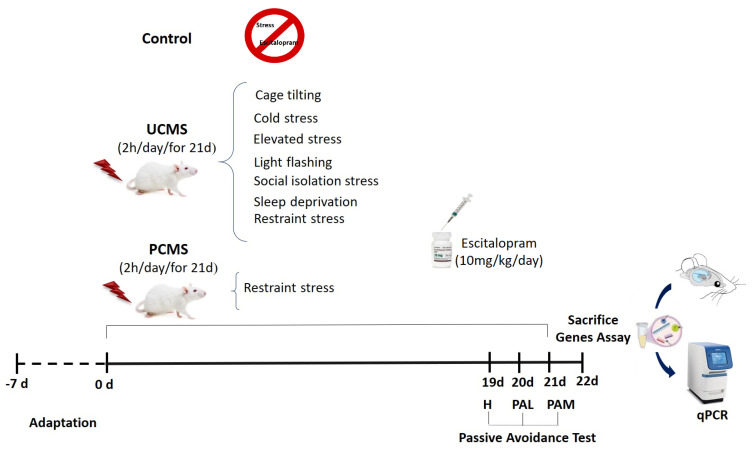
Detailed schematic design of the experimental protocol. Animals were subjected to chronic predictable and unpredictable mild stress (PCMS and UCMS, respectively) and underwent escitalopram administration. Finally, the rats were sacrificed after a passive avoidance test containing habituation (H), passive avoidance learning (PAL), and passive avoidance memory (PAM). In addition, hippocampal gene expression was assayed with quantitative polymerase chain reaction (qPCR).

Forty-nine male rats weighing 200–250 g were obtained from the animal facility at the Isfahan University of Medical Sciences in Iran. The rats were housed in a controlled environment in a 12 h/12 h light/dark cycle; lights were turned on at 7:00 a.m. The humidity was maintained at 55 ± 5%, and the temperature was kept consistent at 23 ± 2 °C. The rats had unlimited access to food and water, except during the stress period. They were given a week to adjust to the animal facility before the experiments began. The Animal Use Ethics Committee of the Isfahan University of Medical Sciences approved all procedures and protocols (IR.MUI.AEC.1401.049). The animals were randomly assigned to seven groups as follows (n = 7): control (Co) group, where the rats did not receive any specific treatment; sham (Sh) group, where the rats were administered equivalent volumes of saline; escitalopram (Esc) group, where the rats were administered 10 mg/kg of escitalopram daily; predictable stress (PSt) group, where the rats underwent exposure to predictable stress daily; unpredictable stress (USt) group, where the rats underwent exposure to unpredictable stress daily; predictable stress with escitalopram (PSt-Esc) group, where the rats underwent exposure to predictable stress and received escitalopram at a 10 mg/kg dose daily; and unpredictable stress with escitalopram (USt-Esc) group, where the rats underwent exposure to unpredictable stress and received escitalopram at a 10 mg/kg dose daily (Figure 1).

### 2.2. Stress Paradigm

Chronic mild stress (2 h/day, 08:00–10:00 a.m.) was administered for 21 consecutive days in both PCMS and UCMS groups [31]. In this study, each rat was confined in a restrainer to induce PCMS [31]. UCMS was induced through various stress models, including restraint (by placing the rats in Plexiglas restrainers), cage-tilting (by positioning each rat in a tilted cage with a 45° slope, while food and water were positioned at the higher end), elevated platform (by positioning the rats on a platform approximately 70 cm above the ground), cold (by placing the rats inside plastic cages at a temperature range of 2–4 °C), sleep deprivation (by placing a cylindrical clay pedestal, measuring 6 cm in diameter and 5 cm in height, on the cage floor opposite the food and water area), isolation (by keeping each rat in a solitary cage), and flashing light (by exposing the rats to a flashing light stimulus, 2.3 V, 30 times per min) stress models [32,33,34,35,36,37,38].

### 2.3. Drug Treatment

After dissolving a 10 mg/kg dose of pure escitalopram oxalate powder (Sobhan-Daru, Rasht, Iran) in sterile 0.9% saline [39], the solution was administered intraperitoneally (i.p.) to both stressed and non-stressed rats for 21 consecutive days. Briefly, 10 mg of pure escitalopram powder was dissolved in 1 cc of saline. Subsequently, the amount of drug for administration was calculated based on the weight of each rat. The injection solutions were given to each rat in a total volume ranging from 0.2 to 0.25 mL. In previous studies on rats, different doses of escitalopram (1–20 mg/kg) were used [40,41]. Most of these studies used doses of 5 mg/kg and below to investigate fear treatment methods. However, for the treatment of stress and depression, higher doses of 10 and 20 mg/kg were used, respectively, as recommended. Therefore, escitalopram at a dose of 10 mg/kg was used for the treatment of stress [20,39,42].

### 2.4. Passive Avoidance Test

The passive avoidance apparatus used in this study was a shuttle box measuring 20 cm × 25 cm × 64 cm. This apparatus was used to assess a range of cognitive functions, such as learning, memory, consolidation, and locomotor activity [43]. The experimental setup consisted of a device with two identical compartments—one light and one dark—fitted with sliding guillotine doors and a grid floor. The experiment was divided into three phases: habituation (without electrical foot shocks on day 19 for 300 s), learning, and memory trials. In the learning trial (with electrical foot shocks on day 20), rats were placed one by one in the light compartment for a duration of 60 s. Subsequently, the guillotine door was raised, allowing the rat to move into the dark compartment. Then, the door was closed. During the learning trial, the rat received a mild electric shock (0.5 mA, 50 V, and lasting 2 s) to its foot via the grid floor. The time taken for the animal to enter the dark compartment before receiving the electrical shock was recorded as the initial latency time. In the third phase as a memory trial (without electrical foot shocks on day 21), the rat was placed in the light compartment again for 300 s. Subsequently, the latency time for entry into the dark compartment was measured after a day, with a maximum delay of 300 s, and it was considered as memory. The difference between the initial latency time and the latency after 1 day was considered indicative of the learning process observed in the experiment [43]. The total dark stay (DS) time was considered to be either for memory consolidation or for the storage of new information in the memory trial [44]. Additionally, the frequency of entries into the dark compartment during the memory trial was recorded as a measure of locomotor activity [43,45].

### 2.5. Gene Expression Assessment

On the 22nd day of the experiment, the rats were given anesthesia through an intraperitoneal injection of urethane (1.5 g/kg; Sigma-Aldrich Chemical Co., St. Louis, MO, USA), and the subjects were euthanized between 16:00 and 18:00 by decapitation. After decapitation and brain removal, the hippocampi were quickly dissected on dry ice. The left hippocampus was separately extracted for *BDNF* and *BCL-2* gene expression assessment. The real-time polymerase chain reaction (PCR) method was used to assess the levels of gene expression for *BDNF* and *BCL-2* within the dissected left hippocampus. As mentioned earlier, the *BDNF* gene regulates apoptotic cell death in the hippocampus by promoting neurogenesis, while *BCL-2* exerts an anti-apoptotic effect in this region [46,47]. Hippocampal tissue total RNA was extracted using the Hybrid-R™ kit (Gene All Biotechnology Co., Seoul, Korea) following the manufacturer’s guidelines. This technique involves breaking down cells with a chaotropic salt, attaching RNA to silica-based membranes, rinsing the RNA with a wash buffer containing ethanol, and, ultimately, extracting purified RNA using RNase-free deionized distilled water (ddH_2_O). Afterward, the quality of messenger RNA (mRNA) was assessed through gel electrophoresis, and the RNA concentration was determined using Nano Drop at a wavelength of 260 nm. In the reverse transcription process, 5 ng of total RNA was used to create complementary DNA with a random hexamer’s primers using the Reverta-L kit (Amplisens, Moscow, Russia), following the manufacturer’s instructions. Real-time PCR was conducted using the Step One Plus real-time PCR System from Applied Biosystems. Real Q Plus 2x Master Mix Green with high ROX™ (Ampliqon) was used, along with the primer sequences specified in Table 1 of this study. Beta-actin (ACTB) served as the internal control for standardizing RNA input. The real-time PCR cycle parameters included an initial denaturation phase at 95 °C for 1 min, followed by denaturation at 95 °C for 15 s and annealing/extension at 60 °C for 60 s. The Ct value, which represents the cycle number at which fluorescence exceeds a predetermined threshold, was determined. The fold change was computed using the 2^−ΔΔCt^ method, indicating the alteration in the treated experimental group compared to the respective control group after normalization to the ACTB endogenous control [25,46].

### 2.6. Statistical Analysis

The data were expressed as the mean ± standard error of the mean (SEM). Statistical analyses comprised a one-way Analysis of Variance (ANOVA) test, followed by Tukey’s post hoc test for between-group comparison. Paired-sample t-tests were employed for within-group comparisons between initial latency and latency after 1 day in the passive avoidance test. The criterion for statistical significance was set at *p* < 0.05. All statistical analyses were performed utilizing IBM SPSS Statistics, version 26 (SPSS Inc. Chicago, IL, USA).

## 3. Results

The comparison of data between the control and sham groups did not reveal any statistically significant differences. Consequently, the findings of the experimental groups were compared with those of the control group.

### 3.1. Passive Avoidance Test 

**Figure 2:**
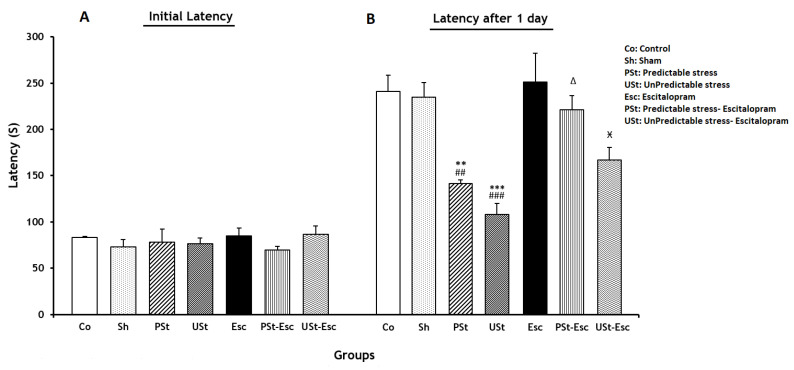
(**A**) Initial latency and (**B**) latency after 1 day to entrance into the dark compartment of the passive avoidance (PA) apparatus for all experimental groups (between groups) (n = 7). The results are shown as the mean ± SEM (one-way ANOVA followed by Tukey’s post hoc test). ** *p* < 0.01 and *** *p* < 0.001 compared to the Co group; ^##^ *p* < 0.01 and ^###^ *p* < 0.001 compared to the Sh group; ^Δ^ *p* < 0.05 compared to the PSt group; ^Ӿ^ *p* < 0.05 compared to the Esc group.

There were no significant differences in the initial latency across the experimental groups, as illustrated in Figure 2A. Additionally, latency following 1 day exhibited a significant decrease in the PSt and USt groups in comparison to the Co group (*p* < 0.01 and *p* < 0.001, respectively). Additionally, latency after 1 day showed a significant enhancement in the PSt-Esc group compared to the PSt group (*p <* 0.05). However, latency after 1 day significantly decreased in the USt-Esc group compared to the Esc group (*p <* 0.05) (Figure 2B).

**Figure 3:**
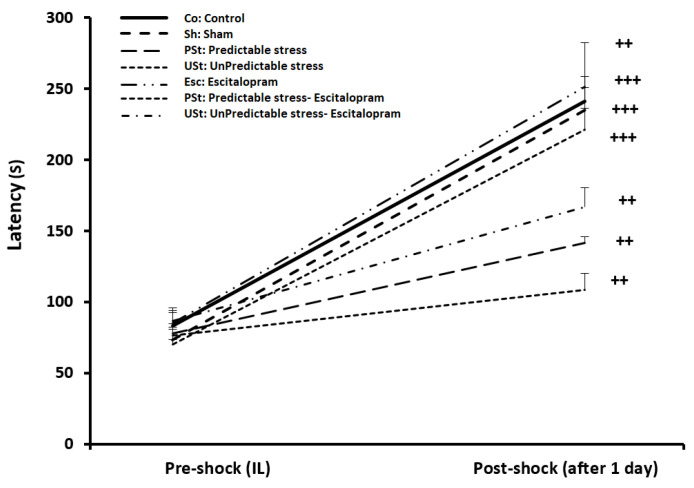
Pre-shock (initial latency, IL) and post-shock (latency after 1 day) to entrance into the dark compartment of the passive avoidance (PA) apparatus before and after the foot shock (within groups) (n = 7). The results are shown as the mean ± SEM. ^++^ *p* < 0.01 and ^+++^ *p* < 0.001 Initial latency relative to the latency after 1 day.

The study conducted an analysis on the initial latency and latency after 1 day using a paired-sample *t*-test, as depicted in Figure 3. The purpose was to assess any alterations in latency within the group. The results indicated significant discrepancies between the initial latency and latency after 1 day, as determined by the PA test in the Co, Sh, Esc, and PSt-Esc groups (all *p* < 0.001), as well as the PSt, USt, and USt-Esc groups (all *p* < 0.01). The findings suggested that learning took place across all groups, although in varying degrees. Notably, the USt group exhibited the lowest level of learning, while the Esc group demonstrated the highest level of learning (Figure 3).

**Figure 4:**
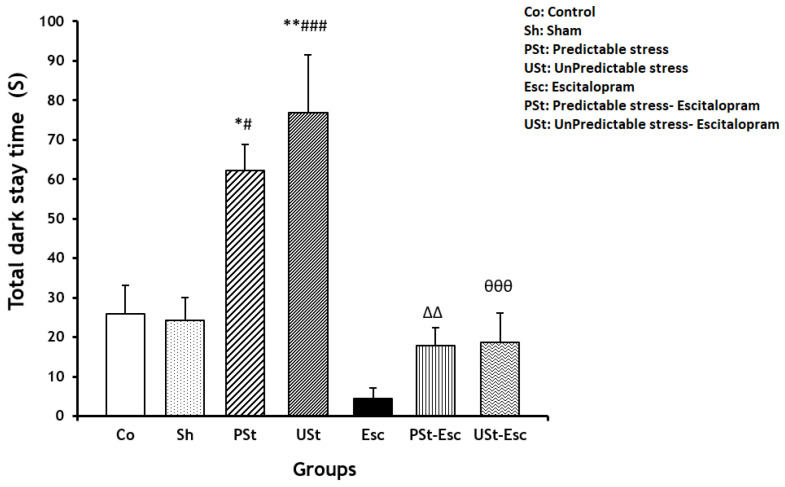
Total dark stay time in the passive avoidance apparatus for all experimental groups (n = 7). The results are shown as the mean ± SEM (one-way ANOVA followed by Tukey’s post hoc test). * *p* < 0.05 and ** *p* < 0.01 compared to the Co group; ^#^ *p* < 0.05 and ^###^ *p* < 0.001 compared to the Sh group; ^ΔΔ^ *p* < 0.01 compared to the PSt group; ^өөө^ *p* < 0.001 compared to the USt group.

The total dark stay time in the PSt and USt groups was significantly higher than that in the Co group (*p* < 0.05 and *p* < 0.01, respectively). However, the total dark stay time was significantly lower in the PSt-Esc and USt-Esc groups compared to their stress-related groups (*p* < 0.01 and *p* < 0.001, respectively). Treatment with escitalopram improved memory consolidation in the PSt-Esc and USt-Esc groups (Figure 4).

**Figure 5:**
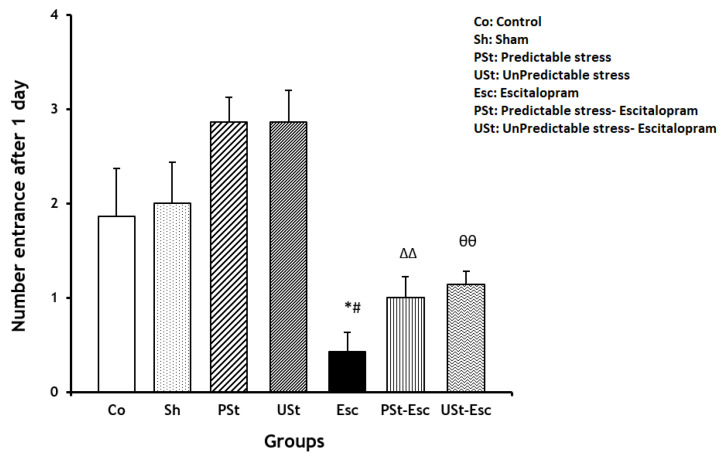
The number of entries into the dark compartment of the passive avoidance apparatus for all the groups 1 day after receiving the foot shock (n = 7). The results are shown as the mean ± SEM (one-way ANOVA followed by Tukey’s post hoc test). * *p* < 0.05 compared to the Co group; ^#^ *p* < 0.05 compared to the Sh group; ^ΔΔ^ *p* < 0.01 compared to the PSt group; ^өө^ *p* < 0.01 compared to the USt group.

There were no statistically significant differences observed in the quantity of entries into the dark compartment between the PSt and USt groups in comparison to the Co group. The number of entries into the dark compartment was significantly lower in the Esc group than in the Co group (*p* < 0.05). In addition, the number of entries into the dark compartment decreased significantly in the PSt-Esc and USt-Esc groups compared to their respective stress-related groups (both *p* < 0.01, respectively). This indicated reduced locomotor activity as a result of treatment with escitalopram under chronic mild stress (Figure 5).

### 3.2. Hippocampal BDNF and BCL-2 Gene Expressions

**Figure 6:**
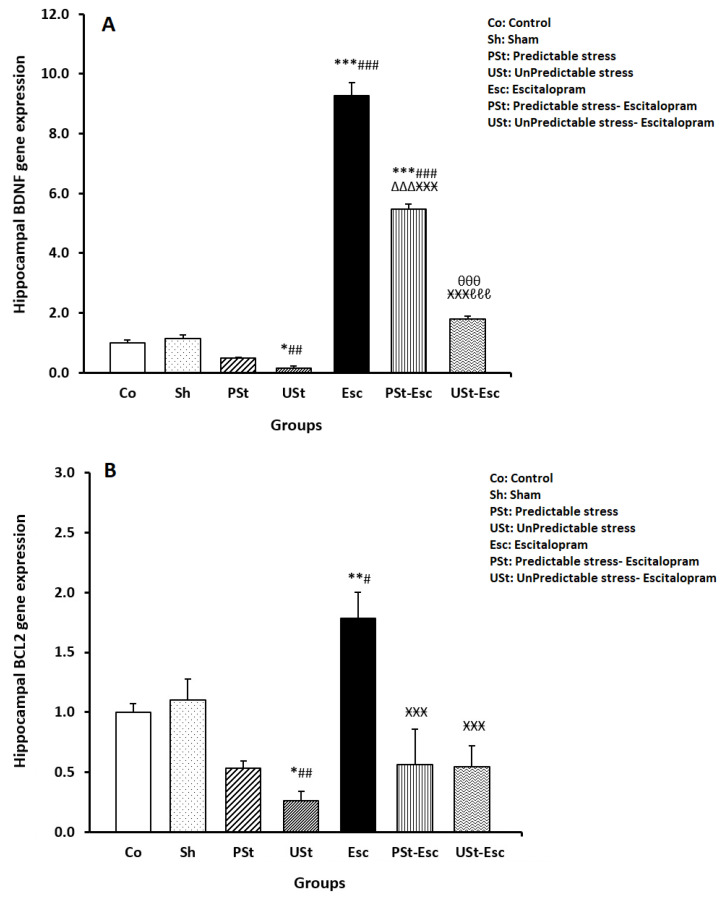
Hippocampal (**A**) *BDNF* and (**B**) *BCL-2* gene expressions measured by RT-qPCR (n = 7). The results are shown as the mean ± SEM (one-way ANOVA followed by Tukey’s post hoc test). * *p* < 0.05, ** *p* < 0.01, and *** *p* < 0.001 compared to the Co group; ^#^ *p* < 0.05, ^##^ *p* < 0.01, and ^###^ *p* < 0.001 compared to the Sh group; ^ΔΔΔ^ *p* < 0.001 compared to the PSt group; ^ӾӾӾ^ *p* < 0.001 compared to the Esc group; ^θθθ^ *p* < 0.001 compared to the USt group; and ^ℓℓℓ^ *p* < 0.001 compared to the PSt-Esc group.

As seen in Figure 6A, the expression of *BDNF* mRNA significantly decreased in the USt group compared to the Co group (*p* < 0.05). The *BDNF* mRNA expression significantly increased in the Esc and PSt-Esc groups compared to the Co group (both *p* < 0.001). The expression of *BDNF* significantly increased in the PSt-Esc group compared to the PSt group (*p* < 0.001) but decreased compared to the Esc group (*p* < 0.001). Furthermore, *BDNF* expression significantly increased in the USt-Esc group compared to the USt group (*p* < 0.001) but decreased compared to the Esc and PSt-Esc groups (both *p* < 0.001) (Figure 6A).

As shown in Figure 6B, the expression of *BCL-2* mRNA significantly decreased in the USt group compared to the Co group (*p* < 0.05), whereas there was a significant increase in the Esc group compared to the Co group (*p* < 0.01). In addition, *BCL-2* mRNA expression significantly decreased in the PSt-Esc and USt-Esc groups compared to the Esc group (both *p* < 0.001) (Figure 6B).

## 4. Discussion

This study was designed to investigate the protective effects of long-term escitalopram administration on memory and hippocampal *BDNF* and *BCL-2* gene expressions in rats exposed to predictable and unpredictable chronic mild stress.

According to the current findings, learning occurred in all experimental groups at varying levels, with a low level of learning observed in the UCMS subjects. In support of the findings, although stress has the potential to accelerate the development and intensity of cognitive deficits, it is possible that learning can still occur, even in stressful environments, in rodents [3,48]. Chronic stress is an unavoidable occurrence in daily life that has detrimental effects on learning and cognition through various mechanisms. These mechanisms include changes in brain cell properties, alterations in the activity of the HPA axis, modifications in neural plasticity, and the release of neurotransmitters, such as serotonin, GABA, glutamate, and acetylcholine, as well as the involvement of receptor subtypes [2,6,19,49,50]. In addition, it has been reported that SSRI medications, such as escitalopram, can enhance learning and reverse learning impairments by increasing serotonin levels in the brain [51,52], although escitalopram may seem to be implicated in a paradoxical reversal learning paradigm in rodents [53].

Based on the total-dark-stay-time findings, exposure to both PCMS and UCMS conditions severely impairs memory and memory consolidation. It has been demonstrated that chronic stress can impair cognitive functions, including memory and memory consolidation [54,55]. Different mechanisms have been proposed to explain the changes induced by stress, including alterations in biochemical substances; hormones, such as glucocorticoids; neurotransmitters; oxidative stress; and *BDNF* and *BCL-2* levels in rodents [27,30]. Additionally, morphological changes, such as alterations in dendritic spine density in specific brain regions, have been observed under stressful conditions [56]. In general, chronic mild stress (both predictable and unpredictable) has complex effects on memory, including impairing, neutral, and facilitating effects [8,57].However, the administration of escitalopram reversed memory impairments only in the PCMS subjects, whereas memory consolidation was reversed under both PCMS and UCMS conditions compared to normal conditions. Nevertheless, there are different opinions on the efficacy of escitalopram in brain cognition. Similarly, a previous study has shown that escitalopram may improve stress-induced memory deficits under chronic stress [39]. In addition, escitalopram has been shown to improve various types of memory in subjects experiencing predictable and unpredictable chronic stress [21,39]. It is possible that escitalopram reverses memory dysfunction by activating certain signaling pathways to alter BDNF [21], serotonin [14], and dopamine levels [11]; promote VEGF-mediated angiogenesis [58]; enhance antioxidant defense [21]; stimulate brain plasticity [59]; and increase metabolic capacity [21]. Conversely, there have been reports indicating that escitalopram has the potential to improve memory consolidation under chronic stress conditions [21,39]. In contrast, Jensen et al. (2014) examined the adverse effects of escitalopram on cognitive abilities [60]. Nevertheless, there were some positive and negative effects and even no effect on cognitive improvement from using escitalopram at a dose of 10 mg/kg in rats [61]. The efficacy of escitalopram on cognitive functions may depend on the type and dose of medication, the duration of treatment, and the type and duration of stress [39]. This study suggests that higher doses of escitalopram may be necessary to address memory deficits caused by UCMS.

In this study, both PCMS and UCMS did not alter locomotor activity. There are conflicting findings regarding the impact of stress on locomotor activity. For example, some studies have shown a decrease in locomotor activity, an enhancement of locomotor activity, and no stress-related effects. These findings are discussed in various studies. Furthermore, the administration of escitalopram alone reduces locomotor activity in normal subjects. A decrease in locomotor activity as a result of escitalopram application has been reported in normal situations in rodents [39,62]. Escitalopram has also been shown to increase locomotor activity [42,63]. Therefore, the level of responsiveness to locomotor activity may differ depending on the dose of escitalopram, the type of behavioral test, and/or the experimental protocol [63]. However, in this study, locomotor activity decreased with escitalopram administration under both PCMS and UCMS conditions. It has been reported that escitalopram reduces locomotor activity in unpredictable chronic stress [62]. According to Lin et al. (2016), the administration of escitalopram at a dosage of 10 mg/kg/day did not impact the motor activity of rats, even when they were subjected to stress conditions [64]. In contrast, the administration of escitalopram at a dosage of 10 mg/kg/day resulted in elevated levels of locomotor activity in response to stressful conditions [42]. Therefore, conflicting views have been presented regarding the effects of escitalopram on motor activity. These views are influenced by factors such as the type and duration of medication administration, the type and duration of stress, variations in gene expression, and the specific behavioral tests used to assess motor activity [9,47,65]. In addition, changes in dopamine levels and/or the inhibition of dopamine neuron firing in certain brain regions may be responsible for these conflicting views [66]. 

In this research, hippocampal *BDNF* and *BCL-2* mRNA gene expressions were reduced only under UCMS conditions but not in PCMS subjects, possibly due to the more destructive effects of UCMS conditions. It seems that PCMS and UCMS have fundamental differences in hippocampal gene expressions. Salari et al. (2023) reported that UCMS can cause neuronal apoptosis by downregulating neurotrophic factors, such as *BDNF*, and upregulating *BCL-2* [67]. Therefore, chronic mild stress can lead to neuronal apoptosis and a reduction in neurogenesis in the hippocampus [68,69,70]. PCMS and UCMS probably induce a variety of intracellular signaling pathways and gene expressions [30]. Furthermore, PCMS and UCMS reduce cell turnover by modulating apoptosis, slowing down neurogenesis via stress hormones [71,72], and inducing epigenetic changes [36,37]. Consequently, chronic stress exposure results in a decrease in hippocampal *BDNF* and *BCL-2* levels, which, in turn, causes neuronal toxicity, apoptosis, and inflammation [30]. These factors are known to significantly contribute to memory and cognitive deficits [73]. Various types of stress have detrimental effects on brain functions, stress biomarkers, and the expression of *BDNF* and *BCL-2* genes in rats [48,74]. Several studies have indicated that there are no alterations observed in the gene expressions of hippocampal *BDNF* and *BCL-2* mRNA following exposure to chronic stress conditions lasting 7 and 28 days [30,47,75,76]. Finally, chronic mild stress has small and transient effects on the cell number and volume, which can eventually lead to structural abnormalities in stress disorders over time [77]. Changes in gene expression are associated with improvements in mood and anxiety disorders [78]. However, similar to the findings of this study, the administration of escitalopram alone increases both hippocampal *BDNF* and *BCL-2* mRNA gene expressions [21,30]. It seems that escitalopram can affect both hippocampal neurogenesis and anti-apoptosis [21,79]. Another study reported that the administration of escitalopram at a dosage of 10 mg/kg reduces the levels of BDNF protein in the hippocampus of rat brains [61]. Recent gene expression findings have revealed that the administration of escitalopram increased hippocampal *BDNF* mRNA gene expression in rats exposed to both PCMS and UCMS conditions, whereas hippocampal *BCL-2* mRNA gene expression did not change in these subjects. It seems that achieving changes in *BCL-2* mRNA gene expression is more challenging than altering *BDNF* mRNA gene expression under various chronic stress conditions when treated with escitalopram. Therefore, the promotion of neurogenesis is more readily achieved than anti-apoptosis with escitalopram in both PCMS and UCMS conditions. Shen et al. (2019) reported that unpredictable stress reduces the expression of the *BCL-2* mRNA gene in rat limbic structures [80]. In contrast, it has been demonstrated that escitalopram modulates the expression of specific genes, such as *BDNF* and *BCL-2* [21,81]. Han et al. (2020) reported that escitalopram at a dose of 10 mg/kg does not affect important memory-related parameters, such as hippocampal BDNF levels and serotonin receptor expression [82]. This study suggests that higher doses of escitalopram may be required to improve memory deficits caused by UCMS. Overall, it is imperative to conduct additional cellular research, specifically examining more neurogenesis and apoptosis gene expression, as well as the efficacy of hormones and neurotransmitters, such as glucocorticoids, dopamine, and serotonin, and their receptors under PCMS and UCMS conditions.

This study was subject to certain limitations. There was a valid point made about the limitations of the unpredictable chronic mild stress model. The unpredictable chronic mild stress model primarily focuses on stressors and does not fully account for individual differences in resilience to stress. This can be a limitation, especially since research suggests that resilience mechanisms might be more powerful than susceptibility factors. Therefore, it would be valuable to incorporate measures of stress resilience alongside the unpredictable chronic mild stress model in future studies. This would allow for a more nuanced understanding of how stress interacts with individual vulnerability and resilience in contributing to the pathogenesis of depression. Another limitation of this study is the absence of female subjects following a similar protocol. By unraveling the roles of these factors, we can gain a deeper understanding of the fundamental processes underlying brain function. This knowledge will be instrumental in developing new therapeutic approaches.

## 5. Conclusions

To summarize, both predictable and unpredictable chronic mild stress had devastating effects on memory and memory consolidation. In addition, the administration of 10 mg/kg of escitalopram improved memory only in individuals experiencing predictable chronic mild stress. Hippocampal *BDNF* and *BCL-2* mRNA gene expressions were reduced only in UCMS subjects, not in PCMS subjects. This could be attributed to the more detrimental effects of unpredictable stress conditions. It seems that PCMS and UCMS had fundamental differences in hippocampal gene expressions. Hence, at a dosage of 10 mg/kg, escitalopram does not protect against stress-induced memory deficits in unpredictable chronic mild stress. Finally, escitalopram treatment increased only hippocampal *BDNF* mRNA expression under PCMS and UCMS conditions, while hippocampal *BCL-2* mRNA gene expression did not change in these subjects. It appears that escitalopram can modulate *BDNF* mRNA gene expression more easily than *BCL-2* mRNA gene expression under chronic mild stress conditions. Therefore, neurogenesis occurs more conveniently than anti-apoptosis under both PCMS and UCMS conditions with escitalopram.

## Figures and Tables

**Table 1:** The primers used in real-time PCR tests.

Primer	Sequences (5′ to 3′)	
*BCL-2*	Forward	TAACGGAGGCTGGGATGC
	Reverse	TGAGCAGCGTCTTCAGAGA
*BDNF*	Forward	AGAATGAGGGCGTTTGCGTA
	Reverse	CCTGGTGGAACATTGTGGCT
*ACTB*	Forward	AGGCCCCTCTGAACCCTAAG
	Reverse	CCAGAGGCATACAGGGACAA

PCR: polymerase chain reaction; BCL-2: B-cell lymphoma 2; BDNF: brain-derived neurotrophic factor; ACTB: beta-actin.

## Data Availability

Supporting data are available upon responsible request. The data are not publicly available due to privacy.
